# 肺癌日间手术操作流程与临床应用效果分析

**DOI:** 10.3779/j.issn.1009-3419.2020.02.02

**Published:** 2020-02-20

**Authors:** 映显 董, 道君 朱, 国卫 车, 伦旭 刘, 坤 周, 涛 朱, 洪升 马

**Affiliations:** 1 610041 成都，四川大学华西医院胸外科 Department of Thoracic Surgery, West China Hospital, Sichuan University, Chengdu 610041, China; 2 610041 成都，四川大学华西医院麻醉科 Department of Anesthesiology, West China Hospital, Sichuan University, Chengdu 610041, China; 3 610041 成都，四川大学华西医院日间手术中心 Day Surgery Center, West China Hospital, Sichuan University, Chengdu 610041, China

**Keywords:** 日间手术, 住院手术, 加速康复, 肺癌, Day surgery, Inpatient surgery, Enhanced recovery after surgery, Lung neoplasms

## Abstract

**背景与目的:**

日间手术的种类和数量在不断扩大，部分经过选择的肺癌患者进行日间手术，临床效果如何？基于加速康复外科（enhanced recovery after surgery, ERAS）理念和外科微创技术，探索肺癌患者日间手术的操作流程及其临床应用效果。

**方法:**

选取2019年6月-2019年11月四川大学华西胸外科单个医疗组连续收治行肺手术患者150例，最终纳入研究患者48例，其中住院手术（inpatient surgery group, ISG）患者28例和日间手术患者（day surgery group, DSG）20例。分析两组患者平均住院日、住院费用及并发症等。

**结果:**

平均住院日在日间手术组（1 d）显著低于住院手术组（7.7±2.8）d（*P*=0.000）; 平均住院费用在日间手术组（38, 297.3±3408.7）￥显著低于住院手术组（47, 831.1±7376.1）￥（*P*=0.000）。术后总体并发症发生率在日间手术组（5.0%）与住院手术组（3.6%）无统计学差异（*P*=0.812）。术后总体不良反应发生率日间手术组（10.0%）与住院手术组（17.9%）无统计学差异（*P*=0.729）。

**结论:**

经过选择的肺癌患者行日间手术是可行的且能够加速康复。

## Abstract (en)

**Background and objective:**

The types and number of day surgery are increasing, what is the result of day surgery of selected patients with lung cancer? To explore the operation process and clinical effect of day surgery in patients with lung cancer by fusing the concept of enhanced recovery after surgery (ERAS) and minimally invasive surgical techniques.

**Methods:**

A prospective study was planned with the approval of our institutional review board. 153 lung cancer patients who underwent anatomic resection in a single medical group between June 2019 and Nov 2019 were randomized. 20 patients were applied day surgery and 28 patients by inpatient surgery and the average length of stay, average hospital cost, complications and adverse reactions were analysed.

**Results:**

The average hospital day in DSG group (1 d) was significantly shorter than in ISG group (7.7±2.8) d (*P*=0.000). The average hospital cost in DSG group (38, 297.3±3, 408.7)￥ was significantly lower than in ISG group (47, 831.1±7, 376.1)￥ (*P*=0.000). There was no significant difference in the incidence of postoperative complications between the daytime surgery group (5.0%) and the inpatient surgery group (3.6%) (*P*=0.812). The postoperation adverse reactions in DSG (10.0%) and ISG (17.9%) is no difference (*P*=0.72).

**Conclusion:**

Our study showed that the same clinical effect achieved between DSG and ISG, and recover quickly lung cancer patients after day surgery.

微创技术和加速康复外科（enhanced recovery after surgery, ERAS）理念的结合共同促进外科手术向更小创伤和更低风险发展^[1, 2]^，要重新审视外科治疗的临床观念和操作流程，尤其是是否可以使部分住院手术患者日间化管理？外科手术日间化，具有加快病床周转、提高医疗资源使用效率、减少院内感染等优点^[3]^。日间手术在我国已有56种手术常规开展，且已积累了成套的管理经验，但目前仍局限在相对简单的手术。目前对于相对复杂的手术仍需要住院手术，然而随着微创技术和ERAS理念的推广，尤其疼痛和管道管理的改进^[4]^，是否可以使部分（无严重伴随疾病）肺癌患者由住院手术转日间手术呢？国内外胸外科手术日间化探索仍局限于相对简单的手术^[5-7]^，而对于解剖性肺切除术日间化管理相比于住院手术是否安全可行、尚未见报道。我们利用四川大学华西医院日间手术中心平台，进行了肺癌日间手术的操作流程研究和临床应用效果分析，结果如下。

## 1 资料与方法

### 1.1 临床资料

连续分析2019年6月-2019年11月在四川大学华西医院胸外科单个医疗组行肺手术（肺叶或肺段）患者150例。纳入标准：①年龄 < 50岁; ②病理学检查诊断为原发性肺癌; ③手术方式肺段、肺叶（单叶或双叶）切除术+系统淋巴结清扫术; ④临床资料完整且签署知情同意书。排除标准：①病理诊断为转移性肺癌患者或病历资料不完整; ②未签署知情同意书的患者; ③术前接受放化疗或病理最终诊断为良性或转移性肿瘤患者; ④术后出血或持续漏气需要再次手术的患者。最终纳入患者48例，其中日间手术组（day surgery group, DSG）组20例，住院手术组（inpatient surgery group, ISG）28例（筛选流程见图 1）。术后分期采用国际抗癌联盟（Union for International Cancer Control, UICC）（2009）肺癌分期标准。患者临床特征见表 1。

**1:**
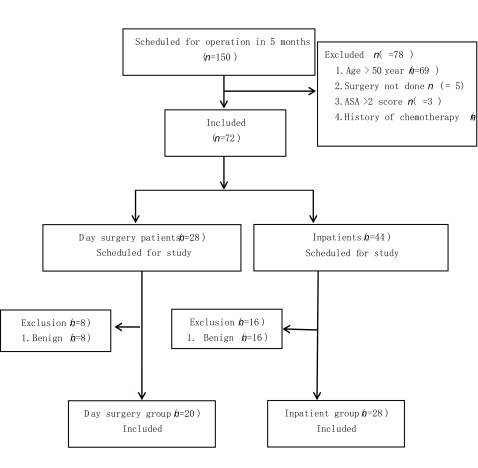
两组患者筛选流程 Study profile for two group cases. ASA: American Society of Anesthesiologists

**1:** 两组患者临床特征 The characteristics of the patients

Index		DSG (*n*=20)	ISG (*n*=28)	*P*
Gender	Male	4	7	0.371
	Female	16	21	
Age (year)	Male	40.1±9.5	43.8±13.2	0.214
	Female	36.3±11.7	38.1±16.7	0.127
Smoking	Yes	2	2	0.400
	No	18	26	
Comorbidity	Yes	2	3	0.260
	No	18	25	
	Hypertension	1	2	0.137
	Diabetes	0	1	
	Coronary heart disease	0	0	
	Hepatitis B	0	0	
	Chronic bronchitis	1	0	
Operation	Lobectomy	10	17	0.121
	Segmentectomy	10	11	
Histology	Adenocarcinoma	20	27	0.277
	Squamouscarinoma	0	1	0.523
	Others	0	0	
TNM stage (2009 UICC)	Ⅰ	20	27	0.327
	Ⅱ	0	1	0.614
Chest drain	Yes	20	28	
	No	0	0	
indwelling urinary catheter	Yes	0	0	
	No	20	28	
Reindwelling urinary catheter	Yes	1	0	
	No	19	28	
TNM: tumor-node-matastasis; UICC: The Union for International Cancer Control.				

### 1.2 方法

#### 1.2.1 手术方法

应用单向式胸腔镜肺叶（1个肺段或2个肺段）切除法+系统淋巴结清扫^[8]^。系统淋巴结清扫，其中肺段切除时，段间平面用切割缝合器进行。左侧必须清扫第5、6、7、8、9、10组淋巴结，右侧包括第2、3、4、7、8、9、10组淋巴结^[9]^。

#### 1.2.2 管道管理

##### 1.2.2.1 尿管管理^[10]^

所有患者围手术期均不留置尿管，入手术室前均排空膀胱，术后回病房，鼓励患者下床自行排尿。当确实存在排尿困难时，护士或家属会采用热敷、床旁站立、温水冲洗外阴、听流水声等方式促进患者排尿，保守处理无效后再置入尿管。

##### 1.2.2.2 引流管管理^[11]^

两组患者均用18 F硅胶球囊导尿管，进行引流。从主操作孔置入（术侧3或4肋间），向球囊中注入15 mL生理盐水并回拉贴紧胸壁，接三腔水封引流瓶，不加用负压吸引。日间手术组：在病人返回病房后（约术后3 h），患者下床活动、且咳嗽时均无漏气，复查胸片后显示胸腔无明显积气、积液，于床旁（术后约6 h）拔除引流管。住院病人组：引流管正常管理，术后24 h，病人咳嗽时若无漏气，且引流液颜色及量均正常，拍片肺复张，且胸腔无积液和积气，拔除引流管。

#### 1.2.3 镇痛管理^[12]^

两组患者围手术期均采用区域肋间神经阻滞麻醉镇痛，术前1 h配制局部神经阻滞液总量200 mL（右美托咪定注射液1 μg/kg、地塞米松10 mg、甲基强的松龙40 mg，硫酸镁注射液0.2 mL、5%碳酸氢钠注射液60 mL、盐酸罗哌卡因注射液400 mg、氯丙嗪12.5 mg），配制液呈白色浑浊液体且有结晶。手术结束时，用针垂直于肋间隙刺入壁层胸膜与肋间内肌间隙，在第3、7、9肋间的手术切口周围进行广泛肋间神经阻滞（涉及第2-10肋间），每个肋间分别注入20 mL局部神经阻滞药液。术后若必要时加用非甾体抗炎药（nonsteroidal antiinflammatory drugs, NSAID）（如帕瑞昔布或氟比洛芬酯注射液，按说明书用），或口服布洛芬缓释胶囊或氨酚羟考酮片（按说明书用）。

#### 1.2.4 术后饮食方案^[13]^

两组患者术后均应用短期中链甘油三酯（medium-chain triglycerides, MCT）食谱; 术后4 h，神志清楚后口服100 mL温开水，无恶心、呕吐不适，6 h-8 h饮用开胃流质250 mL，术后10 h-12 h口服50 g营养粉，兑温水250 mL，术后第1天，营养科订餐，MCT饮食，可喝水，进食水果。

### 1.3 观察指标

#### 1.3.1 住院总费用

住院期间所产生的费用，不包括门诊检查或治疗所产生的费用。

#### 1.3.2 住院治疗费用

包括治疗费、护理费和床位费。

#### 1.3.3 胸腔引流管留时间及引流量

从手术后安置到拔除时间; 术后胸腔总引流量，从胸腔引流管安置到拔除时的总引流量。

#### 1.3.4 术后再置管率

包括尿管再置管率和胸腔引流管再置管率。

#### 1.3.5 乳糜胸

诊断标准：乳糜试验（+）且每天引流量 > 500 mL。

#### 1.3.6 术前等待日

入院当天到术前一天时间（计入院当天，手术当天不计算在内）

#### 1.3.7 术后住院日

手术当天到出院前一天时间（计手术当天，出院当天不计算在内）

#### 1.3.8 平均住院日

入院当天到出院当天时间（计入院当天，出院当天不计算在内）

#### 1.3.9 术后并发症

包括：①胸腔积气：胸部X线片提示胸腔积气 > 30%并再次置管，②胸腔积液：胸片提示积液中到大量; ②出血：术后每小时血性引流液在200 mL以上并持续3 h; ③心律失常：包括心房纤颤、房性/室性期前收缩、阵发性室上性心动过速、室性心动过速; ④痰潴留; ⑤肺部感染：明确的病原学证据、影像学提示肺不张或大片状影、发热、白细胞总数 > 10, 000/mL或15, 000/mL; ⑦持续肺漏气：漏气时间 > 7 d并需要临床干预。

#### 1.3.10 术后不良反应

包括：①严重疼痛：VAS评分≥7分; ②恶心呕吐; ③重度皮下气肿：患者手术切口同侧和对侧胸壁、头面颈部出现皮下气肿; ④呼吸困难; ⑤心悸; ⑥头晕; ⑦谵妄：专科会诊确诊。

### 1.4 统计学方法

统计分析采用SPSS 16.0软件包，计数资料采用实际例数及百分比表示，计量资料采用均数±标准差（Mean±SD）表示，计数资料的比较采用*χ*^2^或MonteCarlo确切概率法进行分析，计量资料比较采用两独立样本的*t*检验。*P* < 0.05为差异有统计学意义。

## 2 结果

### 2.1 两组患者住院日分析

术前住院日和术后住院日在日间手术组（0 d, 1 d）均显著低于住院手术组[(4.5±2.1)d, (3.2±1.5)d]（*P*=0.000, *P*=0.000）; 平均住院日在日间手术组（1 d）显著低于住院手术组（7.7±2.8）d（*P*=0.000）。

### 2.2 两组患者平均医疗费用分析

器械材料费在日间手术组（21, 857.2±2, 848.3）¥和住院手术组（23, 286.7±5, 229.9）¥无统计学差异（*P*=0.231）; 住院药费和治疗费在日间手术组[(1, 766.1±236.7)¥, (1, 050.0±320.3)¥]均显著低于住院手术组[(3, 468.4±772.6)¥, (2, 764.9±1, 154.1)¥]（*P*=0.000, *P*=0.000）。平均住院费用在日间手术组（38, 297.3±3, 408.7）¥显著低于住院手术组（47, 831.1±7, 376.1）¥（*P*=0.000）。

### 2.3 两组患者术后相关并发症分析

日间手术组术后总体并发症发生率（5.0%）较住院手术组（3.6%）高，但无统计学差异（*P*=0.812）。日间手术组再置管发生率（5.0%）高于住院手术组（0%），但无统计学差异（*P*=0.416）; 日间手术组心律失常、胸腔积气、胸腔积液、出血、乳糜胸、声音嘶哑发生率（0%, 0%, 0%, 0%, 0%, 0%）与住院手术组（3.6%, 0%, 0%, 0%, 0%, 0%）相比均无统计学差异（*P*=1.00, *P*=NA, *P*=NA, *P*=NA, *P*=NA, *P*=NA）。见表 2-表 3。

**2:** 两组患者医疗费用分析（Mean±SD） The average medical cost of two groups of patients with lung cancer (Mean±SD)

Index	DSG (*n*=20)	ISG (*n*=28)	*P*
Drug cost (¥)	1, 766.1±236.7	3, 468.4±772.6	0.000
Materials cost (¥)	21, 857.2±2, 848.3	23, 286.7±5, 229.9	0.231
Inpatient treatment cost (¥)	1, 050.0±320.3	2, 764.9±1, 154.1	0.000
Average hospital cost (¥)	38, 297.3±3, 408.7	47, 831.1±7, 376.1	0.000

**3:** 两组患者术后相关并发症分析 The comparision of Postoperative complications between the two groups

Index	DSG (*n*=20)	ISG (*n*=28)	*P*
Aerothorax	0 (0.0%)	0 (0.0%)	NA
Hydrothorax	0 (0.0%)	0 (0.0%)	NA
Homorrhage	0 (0.0%)	0 (0.0%)	NA
Arrhythmia	0 (0.0%)	1 (3.6%)	1.00
Chest drain again	1 (5.0%)	0 (0.0%)	0.416
Lung infection	0 (0.0%)	0 (0.0%)	NA
Chylothorax	0 (0.0%)	0 (0.0%)	NA
Persist air leak	0 (0.0%)	0 (0.0%)	NA
Hoarseness	0 (0.0%)	0 (0.0%)	NA
Total	1 (5.0%)	1 (3.6%)	0.201

### 2.4 两组患者术后不良反应分析

两组患者术后总体不良反应发生率在日间手术组（10.0%）与住院手术组（17.9%）无统计学差异（*P*=0729）。日间手术组恶心、呕吐发生率（10.0%）低于住院手术组（17.9%），但无统计学差异（*P*=0.729）; 日间手术组和住院手术组患者术后均未发生严重疼痛、重度皮下气肿和呼吸困难。见表 4。

**4:** 两组患者术后不良反应分析 The comparision of postoperative adverse reactions between the two groups

	DSG (*n*=20)	ISG (*n*=28)	*P*
Severe pain	0 (0.0%)	0 (0.0%)	NA
Nausea and vomiting	2 (10.0%)	5 (17.9%)	0.729
Severe subcutaneous emphysema	0 (0.0%)	0 (0.0%)	NA
Dyspnea	0 (0.0%)	0 (0.0%)	NA
Total	2 (10.0%)	5 (17.9%)	0.729

## 3 讨论

外科技术、设备的发展体现在微创外科，而理念的更新表现在ERAS，二者的整合能否降低手术难度，使风险相对低的部分手术日间化呢？日间手术在分级诊疗成熟的欧美发达国家已非常普及，占其择期手术的70%以上^[14]^。目前看来，在日间手术开展好的医学中心，从技术上和条件上看是完全有可能的, 。外科手术日间化可以加速病床周转、提高医疗资源使用效率的同时降低医疗费用^[15]^。通过优化肺癌手术患者围手术期管理流程，主要是尽量减少医疗干预，增加医疗服务，保障患者安全的前提下，将更多人文理念的因素整合到治疗中去，就可以加速康复并提高患者就医满意度^[16, 17]^。

胸外科目前开展的日间手术主要集中在手汗症和气胸等相对简单的手术，经选择的部分肺癌患者能否通过日间手术来完成呢，流程如何优化呢，回家康复的风险有什么？需要我们通过临床实践来回答。我们通过前瞻性的研究，分析150例连续收治的肺手术，最终纳入肺癌患者48例，分析20例日间手术和同期28例住院手术，两组患者基线一致。日间手术20例患者均在手术室拔除气管插管，术后2 h进食开胃汤和下床活动，16例患者均在术后6 h左右拔除胸腔引流管; 4例患者于第二天早上将胸腔引流管拔除，其中3例因术后第1天因咳嗽时漏气，1例因术中未安置胸腔引流管，术后4 h出现胸腔积气，再次置入胸腔引流管，均未影响出院; 所有患者术后均未出现严重于疼痛症状，2例患者出现恶心呕吐不良反应。两组患者在术后相关并发症发生率（5% *vs* 3.6%, *P*=0.812）及术后不良反应（10% *vs* 17.9%, *P*=0.729）差异均无统计学意义。日间手术组在住院治疗费和住院总费用均显著低于住院手术组，而材料费在日间手术组（21, 857.2±2, 848.3）¥和住院手术组（23, 286.7±5, 229.9）¥无统计学意义。结果提示肺癌日间手术在经过选择的患者是可行的，且临床效果优于住院手术。

肺癌手术日间化的流程如何优化呢^[18]^？术前精准评估患者，将无严重伴随疾病（风险低）的患者纳入日间手术; 术中缩短麻醉和手术时间（单向式胸腔镜肺叶切除术为主要术式也是为缩短手术时间）; 另外术中减少各种管道的应用：不使用尿管; 胸腔引流管目前采用18号硅胶双腔尿管，既满足引流气体和液体，又不需要切口处用缝线固定，可减轻疼痛及不影响切口愈合^[19]^。术后做好镇痛工作：目前我们主要应用局部阻滞麻醉，减少阿片类药物应用，同时减少不必要的监护，方便患者早期下床活动。48例患者临床应用表明，局部阻滞麻醉镇痛法两组均无严重疼痛发生，且恶心呕吐发生率显著低于同期病房应用镇痛泵的患者^[12]^。1例发生胸腔引流管再置管，是因为术中未放置引流管导致，所以对于术中不放置引流管是否可取，仍需要临床观察。尿管没有因尿潴留而再次置尿管。

20例肺癌日间手术存在样本量小，且是单个医院和医疗组，各种风险能不能充分暴露是研究的问题所在，但也为日间化肺癌手术的开展提供了有益的探索方向，目前需要解决的是如何保障患者安全？现有条件下，充分运用好分级诊疗体系，真正做到手术在医院，康复在社区或家庭，使患者在家庭医生，社区医院，专科医院之间合理流动（分级诊疗）。一是利用好日间手术中心现有的平台优势，手术医生与日间手术中心护士一起进行术前宣教，消除患者对手术的恐惧和担心。二是手术团队医生全程参与术后管理，使意外发生时，患者可以迅速转住院病房处理。三是随访体系畅通（目前主要微信群及随访平台）平台^[20]^。

肺癌日间手术的开展使医院紧张的医疗资源能够最大化，能够更好地为病人提供优质的服务，真正实现了患者利益最大化。
